# Heat stress increases insulin sensitivity in pigs

**DOI:** 10.14814/phy2.12478

**Published:** 2015-08-04

**Authors:** M Victoria Sanz Fernandez, Sara K Stoakes, Mohannad Abuajamieh, Jacob T Seibert, Jay S Johnson, Erin A Horst, Robert P Rhoads, Lance H Baumgard

**Affiliations:** 1 Department of Animal Science, Iowa State UniversityAmes, Iowa; 2 Department of Animal and Poultry Sciences, Virginia TechBlacksburg, Virginia

**Keywords:** Heat Stress, insulin sensitivity, metabolism, pig

## Abstract

Proper insulin homeostasis appears critical for adapting to and surviving a heat load. Further, heat stress (HS) induces phenotypic changes in livestock that suggest an increase in insulin action. The current study objective was to evaluate the effects of HS on whole-body insulin sensitivity. Female pigs (57 ± 4 kg body weight) were subjected to two experimental periods. During period 1, all pigs remained in thermoneutral conditions (TN; 21°C) and were fed ad libitum. During period 2, pigs were exposed to: (i) constant HS conditions (32°C) and fed ad libitum (*n* = 6), or (ii) TN conditions and pair-fed (PFTN; *n* = 6) to eliminate the confounding effects of dissimilar feed intake. A hyperinsulinemic euglycemic clamp (HEC) was conducted on d3 of both periods; and skeletal muscle and adipose tissue biopsies were collected prior to and after an insulin tolerance test (ITT) on d5 of period 2. During the HEC, insulin infusion increased circulating insulin and decreased plasma C-peptide and nonesterified fatty acids, similarly between treatments. From period 1 to 2, the rate of glucose infusion in response to the HEC remained similar in HS pigs while it decreased (36%) in PFTN controls. Prior to the ITT, HS increased (41%) skeletal muscle insulin receptor substrate-1 protein abundance, but did not affect protein kinase B or their phosphorylated forms. In adipose tissue, HS did not alter any of the basal or stimulated measured insulin signaling markers. In summary, HS increases whole-body insulin-stimulated glucose uptake.

## Introduction

Heat stress (HS) is a major environmental hazard for both humans and animals. Despite advances in the understanding of heat-related illnesses, there is no treatment against specific aspects of their pathophysiology, and protocols are limited to generic cooling and rehydration (Leon and Helwig 1985). Therefore, a better understanding of the biological consequences of HS is critical in order to develop effective treatment protocols and mitigation strategies.

Interestingly, diabetic humans and rodents are more susceptible to heat-related illnesses, and exogenous insulin rescues this phenotype (Semenza et al. 1999; Niu et al. 2003). Further, thermal therapy improves insulin sensitivity in diabetic and obese rodents and humans (Hooper 1999; Kokura et al. 2007; Gupte et al. 2009). Moreover, we have previously reported that, despite hypercatabolic hallmarks including marked hypophagia and weight loss, HS increases basal and stimulated circulating insulin and decreases adipose tissue mobilization in a variety of species (Baumgard and Rhoads 2013), including pigs (Pearce et al. 2013a; Sanz Fernandez et al. 2015).

An increase in insulin action might explain the increase in whole-body glucose utilization typically observed during HS (Febbraio 2001). Based on sheer mass, skeletal muscle is likely the main glucose sink during hyperthermia. However, the immune system might also consume a considerable amount of glucose (Greiner et al. 1994; Maciver et al. 2008), as we and others have demonstrated that HS increases plasma lipopolysaccharide (LPS) concentrations, presumably via disrupting the intestinal barrier function (Hall et al. 2001; Pearce et al. 2013b).

Collectively, this suggests that changes in energetic metabolism, specifically insulin homeostasis, might be critical for successfully adapting to and ultimately surviving HS. Our previous attempts to determine insulin sensitivity in HS ruminants and pigs utilizing glucose and insulin tolerance tests are not conclusive (Baumgard and Rhoads 2013) and it is not clear whether this variability is a technical issue or biology based. Thus, the objective of the current study was to determine the effects of HS on insulin sensitivity utilizing the hyperinsulinemic euglycemic clamp: the gold standard technique to determine whole-body insulin action. We hypothesized that HS would increase insulin sensitivity and this altered energetic status would be independent of reduced feed intake.

## Materials and Methods

### Animals and experimental design

Iowa State University Institutional Animal Care and Use Committee approved all procedures involving animals, which took place at the Iowa State University Zumwalt Station Climate Change research facility. The study was divided into two experimental periods. During period 1 (5 day in length), 12 crossbred prepuberal female pigs (57 ± 4 kg body weight) were exposed to thermoneutral conditions (TN; 21.4 ± 0.6°C, 23 ± 3% humidity, 62 ± 1 temperature–humidity index) and fed ad libitum. During period 2 (5 day in length), pigs were randomly assigned to one of two environmental treatments: (i) constant HS conditions (31.6 ± 0.4°C, 17 ± 9% humidity, 73 ± 1 temperature–humidity index) and fed ad libitum (*n* = 6), or (ii) TN conditions and pair-fed (PFTN; *n* = 6) to their HS counterparts, to eliminate the confounding effect of dissimilar nutrient intake. As-fed period 1 daily feed intake (FI) was averaged for each pig and used as a baseline. For each HS pig, the decrease in FI during period 2 was calculated as the percentage of FI reduction relative to period 1 for each day of HS exposure. This percentage of FI reduction was averaged for all the HS pigs per day of exposure and applied individually to the baseline of each PFTN pig. The calculated amount of feed was offered to the PFTN pigs three times daily (∼0800, 1400, and 2100 h) in an attempt to minimize postprandial shifts in metabolism. All pigs were fed a standard industry diet consisting mainly of corn and soybean meal formulated to meet or exceed the nutrient requirements (National Research Council, 2012). Pigs were individually housed in metabolic crates in one of six environmental chambers with a 12 h:12 h light–dark cycle. Ambient temperature was controlled but humidity was not governed and both parameters were recorded every 5 min by a data logger (Lascar EL-USB-2-LCD, Erie, PA) in each chamber. Rectal temperature was measured with a digital thermometer (ReliOn, Waukegan, IL), respiration rate was determined by counting flank movements, and both indices were recorded twice daily (0600 and 2200 h) and condensed into daily averages. Body weight was collected at the beginning and at the end of the study.

### Daily blood sampling

On day 1 of period 1, indwelling catheters were surgically implanted in both jugular veins while pigs were anesthetized (tiletamine/zolazepam, ketamine, and xylazine mixture); using a percutaneous technique as previously described (Sanz Fernandez et al. 2015). From day 4 of period 1, daily blood samples were obtained at 0800 h after a 2 h fast into disposable glass tubes containing 250 U of sodium heparin that were immediately placed on ice. Plasma was harvested by centrifugation at 1300 × *g* and stored at −80°C.

### Hyperinsulinemic euglycemic clamp

A hyperinsulinemic euglycemic clamp (HEC) was performed after an overnight fast on day 3 of period 1 and day 3 of period 2, in 6 HS pigs and 4 PFTN pigs (catheter dysfunction occurred in 2 PFTN pigs). During the HEC, pigs were constantly infused with 0.6 mU min^−1^˙kg BW^−1^ insulin [expected to be the half maximal effective concentration for the glucose rate of infusion based on (Wray-Cahen et al. 2012)] at 12 mL/h for 3 h with a syringe pump (NE-300, New Era Pump Systems,Inc., Farmingdale, NY). Porcine insulin (29 U/mg; Sigma-Aldrich, St. Louis, MO) was diluted in 0.1 N HCl to a 290 U/mL stock. Infusates were prepared for individual pigs by mixing the required insulin from the stock with saline containing 4% of each pig’s serum. Blood samples were obtained at −60, −45, −30, −15 min relative to the initiation of insulin infusion to determine baseline glucose concentrations for each pig. The euglycemic range was established as ±15% of the mean basal glucose content. An insulin priming infusion was initiated at 0 min at 24 mL/h for 10 min, after which the insulin infusion was decreased to 12 mL/h and this rate was maintained constant through the end of the HEC. Blood samples (1 mL) were obtained every 5–10 min and immediately analyzed for glucose concentration utilizing a glucometer (McKensson, San Francisco, CA). Exogenous 50% dextrose (VetOne®, MWI Veterinary Supply, Boise, ID) was delivered with a modular pump (Deltec 3000, Deltec Inc., St. Paul, MN) and its infusion rate was adjusted in order to maintain euglycemia. Blood samples (3 mL) were collected for further analysis every 15–20 min and immediately placed on ice until plasma harvesting.

### Insulin tolerance test

On day 5 of period 2, after an overnight fast, 6 HS and 6 PFTN pigs were anesthetized with the same protocol used for the catheterization surgery. Once anesthetized, subcutaneous adipose tissue (from the cranial dorsum; AT) and skeletal muscle (*longissimus dorsi*, LD) biopsies were obtained surgically, along with a blood sample. After the initial biopsies, an IV insulin bolus was administered at 0.1 U˙kg BW^−1^ (Casu et al. 2010). Contralateral biopsies and blood samples were obtained again 15 min after the insulin bolus. Tissue samples were immediately snap frozen and blood samples were placed on ice until plasma harvesting. After the second biopsy, anesthetized pigs were killed by exsanguination.

### Blood parameter analysis

Plasma glucose and nonesterified fatty acids (NEFA) concentrations were measured enzymatically using commercially available kits (Wako Chemicals Richmond, VA). The intra- and interassay coefficients of variation were 4.5 and 4.6%, and 1.2 and 5.1% for glucose and NEFA, respectively. Plasma insulin and C-peptide concentrations were analyzed using ELISA kits (Mercodia AB, Uppsala, Sweden) following the manufacturer’s instructions. The intra- and interassay coefficients of variation were 8.6 and 10.2%, and 8.8 and 4.3% for insulin and C-peptide, respectively.

### Western blotting

Whole cell protein from LD and AT was extracted in radioimmunoprecipitation assay (RIPA) buffer containing a protease and phosphatase inhibitor cocktail (Halt™, Thermo Scientific, Rockford, IL). Protein concentrations of the extracts were determined by the bicinchoninic acid assay (BCA, Thermo Scientific, Rockford, IL) and samples were diluted to a common concentration in RIPA buffer without inhibitors. Protein abundance of insulin signaling markers were assessed using western blot. Samples were loaded (20-40 *μ*g) in 4-20% precast gradient gels (Lonza, Basel, Switzerland) for SDS-PAGE separation using standard techniques. The separated proteins were transferred from the gel to 0.2 *μ*m pore size nitrocellulose membrane (Biorad, Hercules, CA). The membrane was blocked in 5% nonfat dry milk diluted in tris-buffered saline with 0.1% Tween-20 for 1 h at room temperature and incubated in primary antibody overnight at 4°C. The primary antibodies were specific to insulin substrate-1 (IRS-1; 1:1,000; sc-7200), phospho-IRS-1 (Tyr 632; 1:1,000; ab109543), protein kinase B (Akt; 1:1,000; CST9272), phospho-Akt (Ser 473; 1;1,000; CST9271), and GAPDH (1:10,000; sc-166545). Membranes were then washed and incubated in HRP-conjugated secondary antibody (Anti-rabbit, 1 50 000, Thermo Scientific 31462; and anti-mouse, 1:100 000, CST7076) for 1 h at room temperature. For protein detection, membranes were incubated in Supersignal® West Pico Chemiluminescent Substrate (Thermo Scientific, Rockford, IL) and exposed to film. Bands were quantified using densitometry (Image Lite software 4.0, LI-COR, Lincoln, NE) and relative protein abundance was calculated by normalizing to a common sample included in all gels. Ponceau-S staining of the membrane and GAPDH abundance were used as loading controls.

### Calculations and statistical analysis

In order to eliminate the confounding effect of dissimilar baseline values, the plasma metabolites’ responses to hyperinsulinemia during the HEC were calculated for each individual pig as the difference between each metabolite’s average during the clamped period (i.e., average during hour two and three of the HEC) and their value during the baseline period (i.e., 1 h prior to the HEC). The rate of glucose infusion (ROGI) was calculated per pig and per HEC by averaging the ROGI values when plasma glucose content was within the euglycemic range.

All data were statistically analyzed using SAS version 9.3 (SAS Institute Inc., Cary, NC). Daily temperature indices, production data, and plasma metabolites were analyzed by repeated measures, using PROC MIXED with an autoregressive covariance structure and day of period 2 as the repeated effect. The model included treatment, day, and their interaction as fixed effects; and period 1 values were used as a covariate. The responses to the HEC and the ITT were analyzed by pre-post repeated measures, using PROC MIXED with an unstructured covariance structure and period or time relative to insulin administration (pre-, post-insulin) as the repeated effects, respectively. The models included treatment, period, or time relative to insulin injection, and their interaction as fixed effects.

For each variable in each model, normal distribution of residuals was tested using PROC UNIVARIATE, logarithmic transformation was performed when necessary, and back transformed into the original scale to be reported in the results. Data are reported as least square means and considered significant if *P *≤* *0.05 and a tendency if 0.05 <  *P *≤* *0.10.

## Results

As expected, during period 2, HS pigs had increased rectal temperatures and respiration rates (1.2°C and ∼3 fold, respectively; *P *<* *0.01) compared to PFTN controls (Table1). Overall, HS decreased FI (34%, *P *<* *0.01), and by design, PFTN pigs’ FI was reduced similarly (Table1). Feed intake acutely decreased (40%) at the beginning of period 2, reached its minimum on day 3 (due to overnight fasting in preparation for the HEC), and increased thereafter without recovering to the period 1 FI level (*P *<* *0.01; Table1). By the end of period 2, HS pigs gained more body weight (35%; *P *=* *0.01) than PFTN controls (Table1).

**Table 1:** Effects of heat stress on body temperature indices, feed intake, and body weight

	Day						SEM	*P*		
	P1*	1	2	3	4	5		Trt†	Day	T*D‡
Rectal temperature, °C										
PFTN§	39.02	39.06^a^	38.86^a^	38.82^a^	38.93^a^	38.87^a^	0.16	<0.01	0.61	<0.01
HS**	39.09	39.91^b^	40.23 ^cd^	40.33^d^	40.02^bc^	40.07^bc^				
Respiration rate, bpm										
PFTN	45	40^xy^	42^z^	41^xyz^	34^x^	39^yz^	5	<0.01	0.04	0.27
HS	41	93^xy^	115^z^	108^xyz^	95^x^	114^yz^				
Feed intake, kg										
PFTN	1.88	1.10^y^	1.16^y^	0.96^x^	1.47^z^	1.50^z^	0.07	0.46	<0.01	0.91
HS	1.89	1.16^y^	1.19^y^	0.99^x^	1.57^z^	1.48^z^				
∆BW††, kg										
PFTN						5.2	0.4	0.01		
HS						7.0				

Means with different letters differ (a–d) (*P *≤* *0.05).

Days with different letters differ (x–z) (*P *≤* *0.05).

* Represents period 1 values that were statistically used as covariate.

† Treatment.

‡ Treatment by day interaction.

§ Pair-fed thermoneutral.

** Heat stress.

†† Change in body weight from period 1 to 2.

During period 2, daily changes in basal plasma glucose, insulin, and C-peptide did not differ between treatments; however, there was a day effect (*P *<* *0.05) in all of these parameters as their concentrations decreased (9, 59, and 44% for glucose, insulin, and C-peptide, respectively) on day 3 as expected because of the overnight fast (Table2). There was a day effect (*P *<* *0.01) on the basal insulin to glucose ratio as it was increased (111%) on day 5 compared to day 1 and 3 of period 2 (Table2). At the beginning of period 2, basal plasma NEFA acutely increased for both treatments, peaked on day 3, and sharply decreased thereafter (*P *<* *0.01); but overall, HS pigs had decreased circulating NEFA (46%; *P *<* *0.01) compared to PFTN controls (Table2).

**Table 2:** Effects of heat stress on temporal changes in plasma metabolites

	Day						SEM	*P*		
	P1*	1	2	3	4	5		Trt†	Day	T*D‡
Glucose, mg/dL										
PFTN§	111.6	95.9^y^	96.3^y^	93.3^x^	99.0^y^	98.0^y^	4.3	0.91	0.04	0.42
HS**	108.9	100.3^y^	98.3^y^	85.1^x^	103.4^y^	97.0^y^	3.6			
NEFA††, *μ*Eq/L										
PFTN	70.6	176.5^y^	240.4^y^	253.5^z^	86.7^x^	113.0^x^	27.3	<0.01	<0.01	0.07
HS	71.5	128.1^y^	73.7^y^	169.9^z^	45.4^x^	56.4^x^	23.8			
Insulin, ng/mL										
PFTN	0.130	0.061^y^		0.031^x^		0.124^y^	0.017	0.77	<0.01	0.22
HS	0.155	0.088^y^		0.045^x^		0.099^y^	0.015			
C-peptide, pmol/L										
PFTN	113.6	93.1^y^		61.0^x^		131.1^y^	16.7	0.97	<0.01	0.69
HS	159.9	105.0^y^		63.8^x^		118.3^y^	14.3			
Insulin:glucose‡‡, AU										
PFTN	1.171	0.618^x^		0.383^x^		1.312^y^	0.161	0.71	<0.01	0.27
HS	1.423	0.683^x^		0.484^x^		0.979^y^	0.135			

Days with different letters differ (x–z) (*P *≤* *0.05).

* Represents period 1 values that were statistically used as covariate.

† Treatment.

‡ Treatment by day interaction.

§ Pair-fed thermoneutral.

** Heat stress.

†† Nonesterified fatty acids.

‡‡ Insulin to glucose ratio.

**Figure 1:**
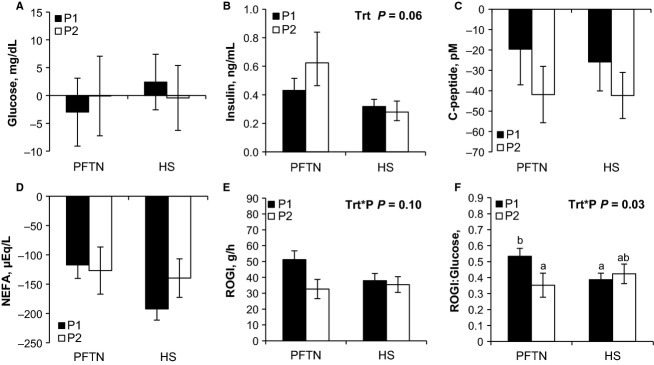
Effects of ad libitum feed intake in constant heat stress conditions (HS; 32°C) and pair-feeding in thermoneutral conditions (PFTN; 20°C) on plasma (A) glucose, (B) insulin, (C) C-peptide, (D) nonesterified fatty acids (NEFA); (E) rate of glucose infusion (ROGI); and (F) ROGI to euglycemic glucose concentration ratio in response to a hyperinsulinemic euglycemic clamp (HEC). Metabolites’ responses were calculated as the difference between the clamped and the baseline periods of the HEC. On period 1 (P1) all pigs were fed ad libitum in thermoneutral conditions. On period 2 (P2) pigs were exposed to either HS or PFTN. ^a, b^Means with different letters differ (*P* ≤ 0.05)

The baseline glucose concentration prior to the HEC decreased similarly from period 1 to 2 (8%; *P *=* *0.03) for both treatments (Table3). Pre-HEC circulating insulin did not differ between treatments or periods (Table3). Overall, baseline C-peptide increased (41%; *P *<* *0.01) in HS pigs compared to PFTN controls, but this difference was similar in periods 1 and 2 (Table3). Prior to the HEC, there was a treatment by period interaction in baseline NEFA (*P *=* *0.04), as it decreased (33%) in HS pigs from period 1 to 2, while it remained unchanged in PFTN controls (Table3). During hyperinsulinemia, euglycemia was maintained as the glucose response was close to 0 and did not differ between treatments or periods (Fig.1A). The overall increase in circulating insulin during the HEC tended to be decreased (43%; *P *=* *0.10) in HS pigs compared to PFTN controls, but this difference was similar in periods 1 and 2 (Fig.1B). As expected, during the HEC, C-peptide and NEFA concentrations decreased, but their response did not differ between treatments or periods (Fig.1C and D). There was a tendency for a treatment by period interaction in ROGI (*P *=* *0.10), as it remained similar in HS pigs from period 1 to 2, while the PFTN pig’s ROGI decreased (36%; Fig.1E). The interaction became significant (*P *=* *0.03) when ROGI was normalized to pre-HEC glucose concentration (Fig.1F).

**Table 3:** Effects of heat stress on the plasma metabolite baselines prior to a hyperinsulinemic euglycemic clamp

	PFTN*			HS†			*P*		
	P‡1	P2	SEM	P1	P2	SEM	Trt§	P	T*P**
Glucose, mg/dL	97	94	5	98	85	4	0.50	0.03	0.11
Insulin, ng/mL	0.035	0.031	0.008	0.051	0.044	0.007	0.12	0.40	0.77
C-peptide, pmol/L	50.9	49.6	6.9	70.7	71.0	5.7	0.01	0.94	0.91
NEFA††, *μ*Eq/L	172.6^a^	212.5^ab^	36.3	282.4^b^	189.3^a^	29.6	0.31	0.35	0.04

Means with different letters differ (a, b) (*P *≤* *0.05).

* Pair-fed thermoneutral.

† Heat stress.

‡ Period.

§ Treatment.

** Treatment by period interaction.

†† Nonesterified fatty acids.

**Figure 2:**
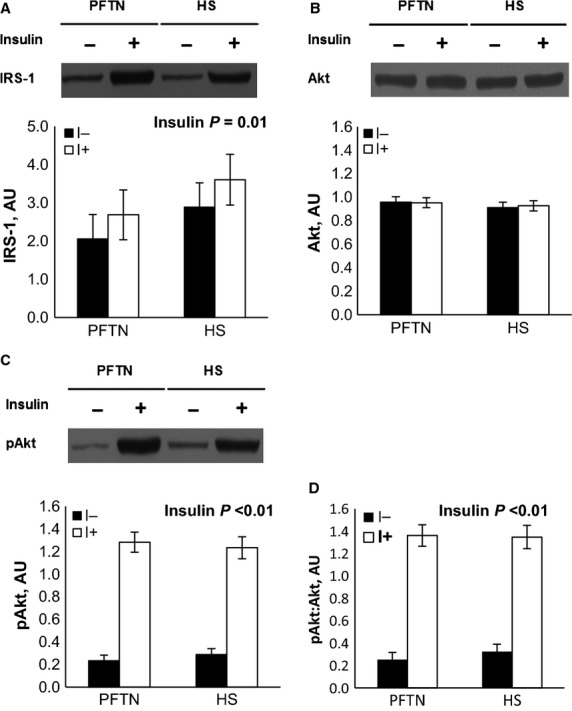
Effects of ad libitum feed intake in constant heat stress conditions (HS; 32°C) and pair-feeding in thermoneutral conditions (PFTN; 20°C) on the adipose tissue protein abundance of (A) insulin receptor substrate-1 (IRS-1), (B) protein kinase B (Akt), (C) phospho-Ser Akt, and (D) phospho-Ser Akt to total Akt ratio in response to an insulin tolerance test. Tissue biopsies were obtained prior (I-) and 15 minutes after (I+) an intravenous insulin dose (0.1 U˙kg BW^−1^).

**Figure 3:**
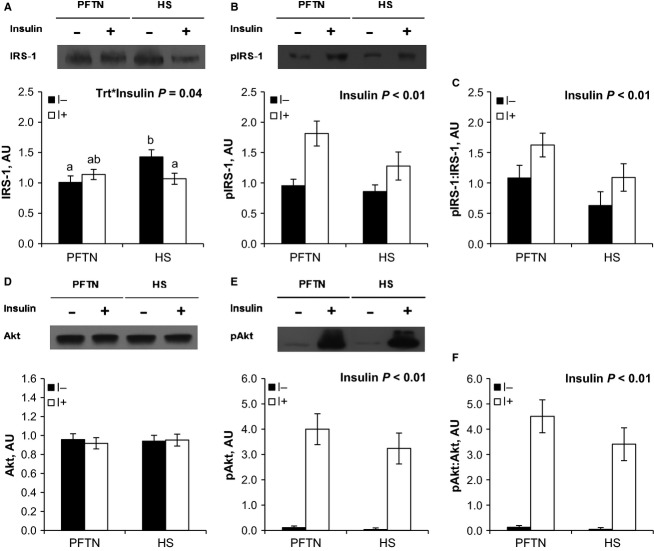
Effects of ad libitum feed intake in constant heat stress conditions (HS; 32°C) and pair-feeding in thermoneutral conditions (PFTN; 20°C) on the skeletal muscle (longissimus dorsi) protein abundance of (A) insulin receptor substrate-1 (IRS-1), (B) phospho-Tyr IRS-1, (C) phospho-Tyr to total IRS-1 ratio, (D) protein kinase B (Akt), (E) phospho-Ser Akt, and (F) phospho-Ser to total Akt ratio in response to an insulin tolerance test. Tissue biopsies were obtained prior (I-) and 15 min after (I+) an intravenous insulin dose (0.1 U kg BW^−1^). ^a, b^Means with different superscripts differ (*P* ≤ 0.05).

During the insulin tolerance test (ITT), insulin administration increased circulating insulin (0.026 vs. 1.749 ng/mL; *P *<* *0.01) and decreased plasma glucose (97 vs. 32 mg/dL; *P *<* *0.01) concentrations similarly between treatments (data not shown). Overall, HS pigs tended to have increased plasma glucose (59 vs. 71 mg/dL; *P *=* *0.08) compared to PFTN controls prior and after the insulin injection (data not shown). The ITT increased AT IRS-1 and phospho-Akt protein abundance, as well as the phosphorylated to total Akt ratio (28%, 5 and 5 fold, respectively; *P *<* *0.01), similarly between treatments (Fig.2A, C, and D). Neither treatment nor insulin had an effect on AT Akt abundance (Fig2B). There was a treatment by time interaction for LD IRS-1 (*P *=* *0.04), as basal protein abundance was increased in HS pigs (42%) compared to PFTN controls, but there were no treatment differences after insulin administration (Fig.3A). Both LD phospho-IRS-1 and phospho-Akt protein abundance, as well as the phosphorylated to total IRS-1 and Akt ratios increased after the ITT (69%, 61-fold, 62%, 62-fold, respectively; *P *<* *0.01), but no treatment differences were detected (Fig.3B, C, E, F). There were no treatment or insulin effects on LD Akt protein abundance (Fig.3D).

## Discussion

Changes in systemic and intracellular energetic metabolism are crucial for successfully adapting to HS. Specifically, proper insulin homeostasis and insulin action appear critical for acclimation and survival to a severe heat load (Semenza et al. 1999; Niu et al. 2003; Baumgard and Rhoads 2013). Interestingly, increasing heat shock protein (HSP) abundance improves insulin sensitivity in various models of diabetes and obesity (Hooper 1999; Kokura et al. 2007; Gupte et al. 2009), which suggests that insulin signaling and heat adaptation are interrelated. Agriculturally relevant species reared under HS exhibit phenotypic changes, like increased adiposity and reduced milk synthesis (Baumgard and Rhoads 2013), that would not be anticipated based on their energetic status. In fact, in the current study, HS pigs gained more weight than PFTN controls which is in agreement with our previous observations (Baumgard and Rhoads 2013; Pearce et al. 2013a), suggesting that the energetics of body weight stasis and tissue accretion are very different between the models. Insulin is a potent lipogenic and antilipolytic signal and thus changes in insulin action may partially explain the body composition differences and milk yield disparity in HS animals.

We have previously reported and corroborated in the current study that, despite marked hypophagia, HS ruminants (Rhoads et al. 2009; Wheelock et al. 2010; Baumgard et al. 2011) and pigs (Pearce et al. 2013a; Sanz Fernandez et al. 2015) do not mobilize as much AT as PFTN counterparts, as demonstrated by a reduction in circulating NEFA and a blunted NEFA response to an epinephrine challenge. A potential explanation for this lack of AT mobilization might be related to changes in insulin homeostasis. We and others have repeatedly reported increased basal insulin in growing and lactating ruminants during HS (Itoh et al. 1998; O’Brien et al. 2010; Wheelock et al. 2010). However, our results in pigs are less conclusive as we have observed increased basal insulin after 7 d of HS (Pearce et al. 2013a), no changes in basal insulin but increased circulating C-peptide in early stages of HS (Sanz Fernandez et al. 2015), and no changes in either basal insulin or C-peptide in the current study. Similarly, the insulin response to a glucose tolerance test was consistently increased in ruminants during HS compared to controls, but glucose disposal was either increased (Wheelock et al. 2010), unchanged (O’Brien et al. 2010), or was blunted (Baumgard et al. 2011). In contrast, pigs had a decreased insulin response and blunted glucose disposal following a glucose tolerance test, but coupling both responses (i.e., into an insulinogenic index) suggested that HS animals required less insulin to stimulate a similar amount of peripheral glucose uptake (Sanz Fernandez et al. 2015). These conflicting results might be due to differences in species [pigs are generally considered more insulin sensitive than ruminants (Brockman and Laarveld 1986)], physiological state, experimental design (e.g., constant vs. cyclical HS), magnitude and length of the heat load, and timing of the metabolic challenge relative to feeding or peak heat load. Regardless, the glucose tolerance test is not an ideal method to determine insulin action as glucose stimulated insulin secretion, insulin clearance, and peripheral insulin sensitivity all contribute to glucose disposal (Muniyappa et al. 2008).

To gain a better appreciation of how HS alters whole-body insulin action we utilized the HEC, which assumes that at the clamped state (i.e., during euglycemia) the amount of exogenous glucose entering the system equals the amount of insulin-stimulated glucose uptake by peripheral tissues (Muniyappa et al. 2008). As anticipated, PFTN pigs experienced a 36% decrease in the ROGI from period 1 to 2, which is a well-described homeorhetic adaptation strategy to a reduced plane of nutrition, where insulin sensitivity is decreased in order to spare glucose for tissues that are obligate glucose utilizers (brain, red blood cells) (Bauman and Currie 1980). However, HS animals maintained a similar ROGI between periods; in other words, insulin sensitivity remained unchanged during hyperthermia despite the large decrease in FI. This treatment difference in ROGI becomes even more noticeable when considering that baseline glucose prior to the period 2 HEC was numerically decreased in HS pigs compared to period 1 and PFTN pigs. Thus, HS induced an apparent increase (when considering period 1 differences between treatments) in the ROGI compared to PFTN conditions despite being clamped at a numerically lower glucose concentration, and this is why differences become more obvious when normalizing to baseline glucose. The increase in whole-body insulin sensitivity agrees with results in lactating cows (Skrzypek et al. 2010), sheep (Achmadi et al. 1993), and the human and rodent literature where thermal therapy improves insulin sensitivity in diabetic and obese individuals (Hooper 1999; Kokura et al. 2007; Gupte et al. 2009).

Nevertheless, interpreting HEC data is based on certain assumptions. The first assumption is that the induced hyperinsulinemia inhibits hepatic glucose output (Girard 2006) equally between treatments. To our knowledge, there are no reports specifically evaluating insulin’s ability to regulate hepatic glucose export during HS. We have previously reported that HS decreases hepatic insulin receptor protein abundance in growing ruminants (O’Brien et al. 2008), which might reduce insulin’s capacity to inhibit hepatic glucose output. In addition, humans exercising in high ambient temperatures have increased hepatic glucose production (Febbraio 2001) and carbohydrate ingestion fails to inhibit this response (Angus et al. 1985). Consequently, insulin may be less effective at reducing hepatic glucose output during HS; a scenario that, if true in the pig, suggests that the treatment differences in insulin mediated whole-body glucose disposal are underestimated and are even greater than the ROGI indicates. The second assumption is that hyperinsulinemia inhibits pancreatic insulin secretion similarly between treatments. This is likely the case in our model as the magnitude of the circulating C-peptide (an indicator of pancreatic insulin secretion (Wallace et al. 2004)) decrease between treatments did not differ.

The increase in the ROGI during HS (compared to the PFTN controls) indicates increased insulin-induced whole-body glucose uptake; however, the tissues responsible for this increased plasma glucose disposal remain unclear. Skeletal muscle is a likely candidate due to its sheer mass and because it is highly responsive to insulin (Kraegen et al. 1985). Additionally, in our model HS increases muscle HSP72 protein abundance (Pearce et al. 2013a), and HSP72 overexpression in skeletal muscle prevents the reduction in insulin signaling in response to a high fat diet, and decreases c-jun amino terminal kinase (JNK) activation (a stress kinase responsible for the inactivation of IRS-1) (Chung et al. 2008). Further, whole-body heat therapy increases glucose uptake and insulin signaling in skeletal muscle, and decreases JNK activation in a HSP72-dependent manner (Gupte et al. 2009). Unexpectedly, we did not detect differences in skeletal muscle insulin signaling other than an increase in basal total IRS-1 protein abundance. One explanation may be that, while we evaluated some key components of the insulin signaling pathway, the increased in glucose uptake during HS is independent of IRS-1 and Akt. In addition, the insulin dose may have overwhelmed the insulin signaling pathway (circulating insulin increased ∼70 fold), preventing us from detecting subtle differences in activation. Further, the fact that pigs were fasted prior to the HEC and the induced hyperinsulinemia itself during the clamp might have altered metabolism and overridden the effects of HS. Overall, further research is required in order to elucidate the contribution of the skeletal muscle to glucose disposal during HS.

Similarly to the skeletal muscle, we did not observe treatment differences in any of the basal or insulin-stimulated AT insulin signaling markers. This is surprising, as insulin is a potent antilipolytic signal and a likely candidate that may explain the lack of AT mobilization (Pearce et al. 2013a; Sanz Fernandez et al. 2015), the increase in fatty acid synthase activity (Pearce et al. 2011), and the decrease in transcript abundance of the adipose triglyceride lipase and the AMPK regulatory subunit genes (Sanz Fernandez et al. 2015) observed in this and other HS experiments. Reasons similar to those discussed for the skeletal muscle might apply to the AT too; however, withstanding the absence of differences in AT insulin signaling, the lack of AT mobilization during HS might be the result of enhanced insulin action by other compounds. For instance, plasma lactate, which is increased in a variety of HS models (Baumgard and Rhoads 2013), mediates insulin antilipolytic effects by interacting with the G protein-coupled receptor 81 (Ahmed et al. 2010). Similarly, heat-induced increase in circulating prolactin (Alamer 2011) might partially mediate the blunted lipolytic response observed during HS (LaPensee et al. 2006; Brandebourg et al. 2007). Moreover, the sharp reduction in thyroid hormones observed during HS (Sanz Fernandez et al. 2015) might also contribute to the lack of AT mobilization as thyroid hormones stimulate lipolysis and NEFA utilization (Pucci et al. 2000). Thus, further research is required to establish whether insulin is involved in or governs AT metabolism during HS.

Another plausible fate of glucose disposal might be the immune system. We and others have demonstrated that HS increases plasma LPS (Hall et al. 2001; Pearce et al. 2013b), presumably due to its deleterious effect on intestinal barrier function and the subsequent increase in intestinal permeability to luminal content (Sanz Fernandez et al. 2014). Interestingly, once activated (e.g., by LPS stimulation) immune cells become obligate glucose utilizers (Maciver et al. 2008), and a substantial glucose sink (Greiner et al. 1994). Elucidating the immune system’s relative contribution to whole-body glucose utilization during HS is of interest.

As mentioned earlier, proper insulin action during HS is critical for survival and adaptation to a heat load as diabetics are more susceptible to heat-related illness/death and insulin administration to diabetic rodents improves survivability to severe HS (Semenza et al. 1999; Niu et al. 2003). This might be due to insulin’s key role in mounting a HSP response and might explain why diabetics have decreased HSP72 expression, correlated with their degree of insulin resistance (Li et al. 2006). Interestingly, strategies intended to increase HSP, including thermal therapy, HSP72 overexpression, and HSP coinducers protect against obesity-induced insulin resistance and improve insulin sensitivity in human and rodent models of diabetes and obesity (Hooper 1999; Kokura et al. 2007; Chung et al. 2008; Gupte et al. 2009). Collectively, these data indicate that there is an interdependent relationship between insulin action and the HSP response, where both are required to successfully adapt to a heat load.

The shift toward glucose utilization observed during HS may also help to explain the importance of increased insulin action in the adaptation to a heat load. For instance, exercising at high temperatures increases skeletal muscle glycogen oxidation at the expense of NEFA (Fink et al. 1975; Febbraio 2001), and increases the respiratory quotient which suggests enhanced glucose oxidation (Hargreaves et al. 1985). The increased reliance in glucose as a fuel during HS might also explain why the liver (a key regulator of plasma glucose) remains responsive to adrenergic signals, while the AT does not (Sanz Fernandez et al. 2015). The mechanism by which HS alters cellular substrate utilization is unknown, but might be related to increased circulating LPS. In skeletal muscle, toll-like receptor 4 activation by LPS favors glucose utilization for ATP production (Frisard et al. 2010). However, in contrast to our HS model, LPS signaling typically induces insulin resistance (e.g., decreased glucose uptake) by activating stress kinases (e.g., JNK and inhibitor of kappa B kinase) in thermoneutral conditions (Liang et al. 2013). Reasons for the apparent inconsistencies between LPS mediated and HS-induced altered muscle bioenergetics are not clear, but enhanced muscle glycogen utilization or increased noninsulin-dependent glucose transport may help explain how glucose’s contribution to cellular ATP production increases despite reduced muscle insulin sensitivity. Regardless, determining if a link exists between heat-induced intestinal barrier dysfunction and the increased carbohydrate utilization observed during HS remains of interest.

In the current study, we demonstrated that HS pigs maintain whole-body insulin sensitivity while PFTN controls have reduced insulin action. Despite its probable contribution to the increase in whole-body glucose uptake, the effects of HS on skeletal muscle insulin signaling warrants further investigation. The mechanism by which HS increases insulin sensitivity and the biological reasons behind an increase in glucose utilization remain unknown. A better understanding of the physiological consequences of HS is critical in order to develop treatment protocols and mitigation strategies for heat-related illnesses.
